# Mechanistic Insights into the Pathogenesis of Polycystic Kidney Disease

**DOI:** 10.3390/cells14151203

**Published:** 2025-08-05

**Authors:** Qasim Al-orjani, Lubna A. Alshriem, Gillian Gallagher, Raghad Buqaileh, Neela Azizi, Wissam AbouAlaiwi

**Affiliations:** 1 Department of Pharmacology and Experimental Therapeutics, University of Toledo, Toledo, OH 43614, USA; qasim.alorjani@rockets.utoledo.edu (Q.A.-o.); lalshri@rockets.utoledo.edu (L.A.A.); gillian.gallagher@rockets.utoledo.edu (G.G.); raghad.buqaileh@rockets.utoledo.edu (R.B.); neela.azizi@rockets.utoledo.edu (N.A.); 2 Department of Clinical Pharmacy, Faculty of Pharmacy, Jordan University of Science and Technology, P.O. Box 3030, Irbid 22110, Jordan

**Keywords:** PKD, ADPKD, cystogenesis, PC1/PC2, cilia, calcium, cAMP, mTOR, Wnt, apoptosis, fibrosis, mitochondria

## Abstract

Autosomal Dominant Polycystic Kidney Disease (ADPKD) is a systemic ciliopathy resulting from loss-of-function mutations in the PKD1 and PKD2 genes, which encode polycystin-1 (PC1) and polycystin-2 (PC2), respectively. PC1 and PC2 regulate mechanosensation, calcium signaling, and key pathways controlling tubular epithelial structure and function. Loss of PC1/PC2 disrupts calcium homeostasis, elevates cAMP, and activates proliferative cascades such as PKA–B-Raf–MEK–ERK, mTOR, and Wnt, driving cystogenesis via epithelial proliferation, impaired apoptosis, fluid secretion, and fibrosis. Recent evidence also implicates novel signaling axes in ADPKD progression including, the Hippo pathway, where dysregulated YAP/TAZ activity enhances c-Myc-mediated proliferation; the stimulator of interferon genes (STING) pathway, which is activated by mitochondrial DNA release and linked to NF-κB-driven inflammation and fibrosis; and the TWEAK/Fn14 pathway, which mediates pro-inflammatory and pro-apoptotic responses via ERK and NF-κB activation in tubular cells. Mitochondrial dysfunction, oxidative stress, and maladaptive extracellular matrix remodeling further exacerbate disease progression. A refined understanding of ADPKD’s complex signaling networks provides a foundation for precision medicine and next-generation therapeutics. This review gathers recent molecular insights and highlights both established and emerging targets to guide targeted treatment strategies in ADPKD.

## 1. Introduction

**Figure 1:**
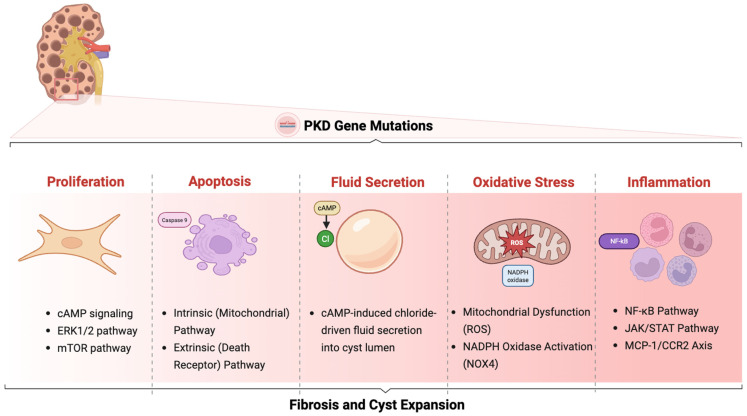
The main pathological mechanisms of Polycystic Kidney Disease (PKD). Mutations in *PKD1/2* genes initiate a cascade of pathological events including abnormal epithelial cell proliferation, dysregulated apoptosis, enhanced fluid secretion, oxidative stress, and chronic inflammation. These processes collectively contribute to progressive renal cyst growth, tubular distortion, and interstitial fibrosis, ultimately impairing kidney function. cAMP: cyclic adenosine monophosphate; ERK: extracellular signal-regulated kinase; mTOR: mammalian target of rapamycin; JAK-STAT: Janus kinase and signal transducer and activator of transcription; NF-κB: Nuclear Factor kappa-enhancer of activated B-cells; ROS: reactive oxygen species; NADPH: nicotinamide adenine dinucleotide phosphate; NOX4: NADPH oxidase 4; MCP-1: monocyte chemoattractant protein-1; CCR2: C-C chemokine receptor type 2. Created with Biorender.com (accessed on 12 June 2025).

Polycystic Kidney Disease (PKD) refers to a group of inherited single-gene disorders typically presenting in adults of all races and ethnicities that affects multiple organ systems such as the liver, heart, spleen, pancreas, and arachnoid membranes [1]. Clinically, PKD manifests as a systemic disorder with extrarenal manifestations, including hepatic cysts, intracranial aneurysms, cardiac valvular abnormalities, and vascular complications. PKD ultimately progresses to end-stage renal disease (ESRD) in over 50% of affected individuals, which are estimated at approximately 600,000 people in the United States and up to 12.5 million worldwide [2,3,4,5]. The morbidity is linked to two forms based on inheritance patterns: Autosomal Dominant Polycystic Kidney Disease (ADPKD), the more frequently occurring type that predominantly involves renal and extrarenal manifestations, and Autosomal Recessive Polycystic Kidney Disease (ARPKD), which is a major cause of illness and death in newborns [6]. At the molecular level, ADPKD arises from germline mutations in the *PKD1* and *PKD2* genes, encoding the transmembrane proteins polycystin-1 (PC1) and polycystin-2 (PC2), respectively [7]. These proteins form a mechanosensory complex localized to the primary cilium and other regions of renal tubular epithelial cells, where they modulate calcium (Ca^+2^) influx and integrate extracellular mechanical and chemical signals to regulate intracellular signaling cascades [8]. Disruption of PC1–PC2 function leads to aberrant Ca^+2^ signaling, elevated cyclic adenosine monophosphate (cAMP) levels, and dysregulation of critical growth and proliferative pathways, including the mammalian target of rapamycin (mTOR), mitogen-activated protein kinase/extracellular signal-regulated kinase (MAPK/ERK), and Wingless-type mouse mammary tumor virus (Wnt)/β-catenin pathways [8,9,10]. These signaling perturbations collectively drive epithelial cell hyperproliferation, cyst expansion, abnormal fluid secretion, and interstitial fibrosis, which are hallmarks of disease progression [11] (Figure 1).

Mutations in *PKD1* and *PKD2* produce distinct clinical phenotypes, with *PKD1* mutations typically resulting in earlier onset and more aggressive disease progression than *PKD2* mutations. These pharmacogenetic differences may influence therapeutic responses, underscoring the importance of considering genotype-specific factors [12]. While current treatments predominantly target shared downstream signaling pathways, future research aimed at mutation-specific therapies could enhance personalized management of ADPKD. Despite substantial advances in the genetic characterization of ADPKD, the precise cellular and molecular mechanisms driving cyst initiation, expansion, and associated renal pathology remain incompletely elucidated [13]. The disease’s complexity arises from a multifaceted network of disrupted signaling cascades, altered ion transport, aberrant epithelial cell behavior, and extracellular matrix remodeling, which are all downstream consequences of dysfunctional polycystin signaling involving both ciliary and non-ciliary pathways [14]. Recent investigations leveraging in vivo models, renal epithelial cell systems, and human-derived organoids have shed light on the interconnected roles of Ca^+2^ and cAMP signaling, metabolic reprogramming, and mechanosensitive feedback loops [15,16]. A comprehensive mechanistic understanding of these dysregulated pathways is essential not only for deciphering the biology of ADPKD, but also for identifying the key molecular inflection points that govern disease onset and progression. In this review, we expand upon established signaling mechanisms by highlighting recent molecular studies that provide deeper insight into their regulation. Additionally, we explore emerging pathways, including the Hippo signaling cascade, the stimulator of interferon genes (STING) pathway, and the fibroblast growth factor-inducible 14 (Fn14) receptor and its ligand TNF-like weak inducer of apoptosis (TWEAK) axis—as well as mitochondrial dysfunction and oxidative stress. We discuss how these signaling alterations contribute to distinct pathophysiological phenotypes such as aberrant cell proliferation, interstitial fibrosis, and apoptosis. By dissecting these core and novel processes, we aim to advance a more integrated and coherent framework for understanding the molecular underpinnings of ADPKD.

## 2. Cellular Mechanisms of Cystogenesis

A hallmark of ADPKD is the progressive development and enlargement of renal cysts, which is a process known as cystogenesis. This phenomenon is primarily caused by genetic mutations in PKD1 and PKD2, coding for the proteins PC1 and PC2, respectively. These proteins localize predominantly at the plasma membrane and primary cilia of epithelial cells lining the renal tubules, where they function as a component of a mechanosensory complex essential for regulating intracellular Ca^+2^ signaling in response to shear stress generated by the flow of fluid through the tubule lumen [17]. Ciliary dysfunction is a hallmark of PKD, playing a central role in multiple cellular abnormalities, including defects in planar cell polarity, impaired cellular differentiation, disrupted signaling pathways, and altered fluid sensing and transport [18]. Alterations in PKD1 or PKD2 lead to the absence or impaired function of polycystins, which compromises ciliary structure and signaling [19]. Consequently, the primary cilium loses its mechanosensory function, leading to impaired Ca^+2^ influx. This disturbance triggers a cascade of intracellular dysregulation, which is characterized by elevated cAMP levels and aberrant activation of key signaling pathways [19]. The consequences of this include abnormally hyper-activated cell proliferation and fluid secretion, which leads to the expansion of renal cysts. In parallel, mis-regulated cell death promotes cyst initiation and expansion, resulting in an imbalance between tubular epithelial cell proliferation and apoptosis, which is one of the pathological features of PKD. Additionally, inflammatory responses and immune cell infiltration contribute to the pathogenic cascade, promoting extracellular matrix remodeling and tubulointerstitial fibrosis, further compromising kidney architecture and function. Furthermore, mitochondrial dysfunction and increased oxidative stress further exacerbate epithelial injury by activating intrinsic apoptotic pathways and amplifying reactive oxygen species (ROS) production that synergistically drive disease progression. Understanding how these interconnected mechanisms govern the cellular and molecular phenotype of ADPKD is critical for elucidating disease progression and informing future therapeutic strategies [20].

### 2.1. Role of Cilia in PKD

The primary cilium is a single, non-motile, microtubule-supported projection that emerges from the surface of almost all renal tubular epithelial cells, except intercalated cells in the collecting duct. It functions as a sensory antenna within the tubule lumen, playing essential roles in mechanosensation, chemosensation, and cell cycle control. It also participates key molecular pathways involved in development, such as those mediated by Hedgehog, Wnt, and platelet-derived growth factor (PDGF). In kidney epithelial cells, the cilium serves as a central hub for signal detection and transduction [21]. Genetic knockout (KO) studies in mice show that the loss of primary cilia leads to abnormal kidney development and the formation of renal cysts, even without abnormalities within the PKD1 or PKD2 genes [22]. A well-known cilia KO model involves deleting the Kif3a gene, which encodes a motor protein subunit required for anterograde intraflagellar transport (IFT). Conditional deletion of Kif3a in renal epithelial cells using tissue-specific promoters like Pax8-Cre or Ksp-Cre causes loss of cilia and rapid cyst formation. These cysts arise due to increased cell proliferation, disrupted planar cell polarity, and excessive fluid secretion [23]. Interestingly, simultaneous knockout of both Pkd1 and Kif3a reduces cyst severity compared to Pkd1 deletion alone. This paradox suggests that when PC1 is absent, the remaining cilium may contribute to pro-cystic signaling. Thus, although cilia loss alone promotes cyst formation, cilia-dependent signaling may further drive disease progression in the presence of dysfunctional polycystins [23]. Similar findings are seen in double knockout models involving Ift88 (also known as Tg737) and Pkd1, supporting the idea that cilia can promote cyst expansion when polycystins signaling is lost. These studies highlight that primary cilia are essential for maintaining epithelial stability, and their loss can initiate or accelerate cystogenesis. Furthermore, when PC1 or PC2 is nonfunctional, cilia may become sources of pathological signaling [24]. IFT88 is a critical component of the IFT machinery and is necessary for cilium assembly and maintenance. It is part of the IFT-B complex and supports the movement of key proteins like tubulin, receptors, and membrane proteins along the ciliary axoneme [25]. Disruption of IFT88 function severely impairs ciliogenesis and causes cystic disease. Loss of IFT88 results in either complete absence or severe shortening of primary cilia [26]. In the Tg737orpk mouse model, which carries a mutation in Ift88, animals develop kidney cysts, abnormal liver morphology, and shortened cilia, which are features similar to human ciliopathies. IFT88 also regulates the trafficking of PC1 and PC2 to the ciliary membrane. When IFT88 is deleted, these proteins fail to localize to cilium, disrupting calcium and mechanosensory signaling. This leads to the activation of several pro-cystic signaling pathways, including mTOR, MAPK, and cAMP-PKA. Importantly, deleting IFT88 can induce PKD-like disease even in the absence of PKD gene mutations, highlighting its upstream and essential role in ciliary signaling [24]. Moreover, the timing of IFT88 deletion is also crucial; embryonic or early postnatal deletion causes more severe disease compared to adult deletion, suggesting a developmental window during which cilia are especially important for kidney patterning [27].

**Figure 2:**
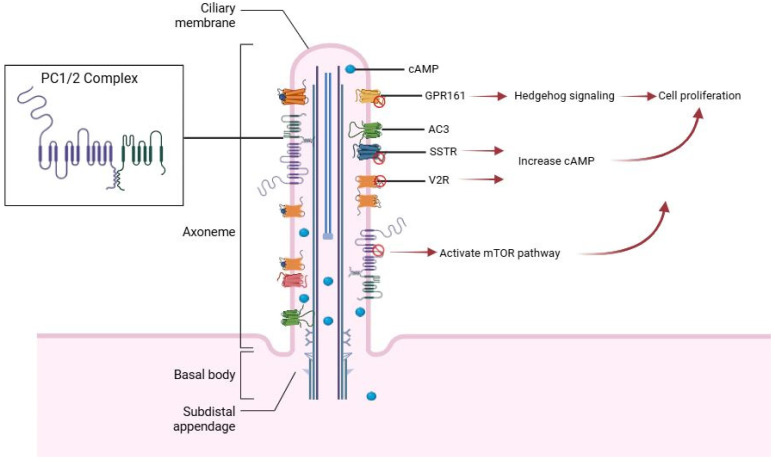
Schematic representation of signaling pathways associated with the primary cilium in renal epithelial cells, highlighting the role of ciliary-localized receptors and proteins in cellular signaling and proliferation. The PC1/2 complex is embedded in the ciliary membrane and plays a mechanosensory role. Key ciliary G protein-coupled receptors (GPCRs) include GPR161, which promotes Hedgehog signaling and drives cell proliferation, and V2R, which increases cAMP levels. AC3 and SSTR also regulate cAMP production. Dysregulation of these pathways can lead to increased cAMP accumulation. Dysfunctional PC1 activates the mTOR pathway, contributing to cystogenesis in Polycystic Kidney Disease. GPR161: G protein-coupled receptor 161; V2R: vasopressin receptor 2; cAMP: cyclic adenosine monophosphate, AC3: adenylyl cyclase 3; SSTR: somatostatin receptor; mTOR: mammalian target of rapamycin. The red prohibition symbol (circle with a diagonal line) denotes a dysfunctional pathway. Created with Biorender.com (accessed on 12 June 2025).

In addition to structural roles, the primary cilium acts as a signaling center enriched with G protein-coupled receptors (GPCRs). GPCRs are a diverse class of membrane proteins that transmit extracellular signals into intracellular responses. A specific subset of GPCRs is known to localize to the primary cilium, where they play critical roles in regulating developmental pathways and maintaining epithelial cell function [28]. In the kidney, the ciliary localization of GPCRs is essential for interpreting mechanical and chemical cues from the tubular lumen. When this signaling becomes disrupted as occurs in PKD, the result is unchecked cellular proliferation, aberrant fluid production, and ultimately cyst formation [29]. The trafficking of GPCRs to the cilium is tightly controlled. These receptors contain cilia-targeting sequences that interact with components of the ciliary trafficking machinery, including BBSome complex and the IFT proteins. When this system is disrupted, GPCRs fail to reach the ciliary membrane, leading to a breakdown in signaling precision. In PKD, such trafficking defects are common and contribute to the mis-regulation of pathways that promote cystogenesis [30]. Several GPCRs have been identified as particularly relevant to PKD. GPR161, a negative regulator of the Hedgehog signaling pathway, typically resides in the cilium and suppresses pathway activation by promoting the processing of Gli transcription factors into their repressor forms. In PKD, however, the loss or mislocalization of GPR161 can result in uncontrolled Hedgehog signaling, promoting excessive proliferation of tubular epithelial cells and contributing to cyst formation [31]. Somatostatin receptors (SSTR1–5) are GPCRs found in the lung and kidney, with prominent localization in renal tubular epithelial cells and glomerular mesangial regions. SSTR1 and SSTR2 are widely distributed along the distal nephron segments and the collecting duct system, while SSTR3–5 are mainly found in the proximal tubules. These receptors bind to the hormone somatostatin, which activates inhibitory G proteins (Gi) to regulate downstream signaling [32,33,34]. Although the role of somatostatin in renal primary cilia is not well understood, one study showed that it reduces cAMP levels by counteracting vasopressin signaling in specific segments of the rat kidney, such as the collecting ducts and thick ascending limb of Henle’s loop [35]. Research has shown that vasopressin V2 receptors (V2Rs) are located on both the basolateral membrane and the primary cilia of renal epithelial cells. When activated by vasopressin, V2R stimulates the Gαs signaling pathway and adenylyl cyclase 6, leading to regulation of water and sodium balance [36]. This occurs mainly through the trafficking and expression of aquaporin-2 in collecting duct principal cells. Vasopressin also promotes the activity of urea transporters (UT-A1 and UT-A3), enhances epithelial sodium channel function in the collecting duct, and upregulates the sodium–potassium–chloride cotransporter at both the expression and functional levels in the thick ascending limb of the loop of Henle [37,38]. The association between GPCRs located on primary cilia and the polycystin proteins, PC1 and PC2, is crucial for regulating calcium signaling in primary cilia, and mutations in Pkd1 or Pkd2 disrupt this complex, promoting cyst formation and kidney dysfunction [39]. PC1 detects mechanical signals and transmits them into cellular responses by interacting with Gα subunits through a unique C-terminal G protein-binding domain. In addition, PC1 contains a conserved GPCR proteolysis site (GPS) within a GAIN domain near its N-terminus, which helps maintain epithelial structure, while its C-terminus interacts with PC2 to regulate ions and calcium signaling; however, its precise role in primary cilia remains unclear [40,41]. Moreover, PC1 suppresses mTOR signaling by interacting with tuberin, and impaired GPCR signaling can disrupt this regulation, resulting in hyperactive mTOR and enhanced cyst development [42] (Figure 2). Collectively, these findings underscore the critical role of primary cilia and cilia-localized GPCR signaling in preserving the functional integrity of renal epithelial cells. When this ciliary signaling system is impaired, whether through structural defects, trafficking failures, or impaired polycystin function, it can initiate or accelerate cystogenesis, contributing to the pathophysiology of PKD.

### 2.2. Cell Proliferation

Cell proliferation is the fundamental process by which cell populations expand, demanding the coordinated growth and division of cells. This crucial and tightly controlled intracellular mechanism underlies the development and maintenance of nearly all tissues in the body. Cell proliferation is regulated by cell cycle control mechanisms and signaling pathways involving protein kinases [43]. The cell cycle consists of four stages (G_0_/G_1_, S, G_2_, and M), with cyclin-dependent kinases (CDKs) playing a central role through phosphorylation of target proteins [44]. The significant enlargement of cysts in the kidneys is a defining characteristic of ADPKD [45]. This expansion is primarily driven by the increased proliferation of the tubular epithelial cells that line these cysts. In healthy kidneys, there is a carefully regulated balance between new cells created through proliferation and old or damaged cells removed through apoptosis (programmed cell death). This balance ensures tissue homeostasis [46]. However, in the kidneys of ADPKD patients and animal models, cell proliferation occurs more frequently than apoptosis. This imbalance between cell growth and cell death contributes significantly to the initiation and progression of cyst formation [47].

Both PC1 and PC2 interact with the inositol 1,4,5-trisphosphate receptor (IP3R) located in the endoplasmic reticulum (ER) to modulate intracellular Ca^+2^ levels [48]. PC2 facilitates Ca^+2^ release from the ER by enhancing IP3R activity, whereas PC1 opposes this action by diminishing the PC2–IP3R interaction through a mechanism that involves stromal interaction molecule 1 (STIM1) and activation of the PI3K/Akt signaling pathway [49]. Studies in Madin-Darby Canine Kidney (MDCK) cells have demonstrated that PC1 plays a crucial role in regulating intracellular Ca^+2^ dynamics through modulation of the PC2–IP3R–STIM1 signaling complex [50]. In cells expressing PC1, ATP treatment-induced Ca^+2^ release was reduced compared to control cells, correlating with a decreased interaction between endogenous PC2 and IP3R, and an enhanced association between IP3R and STIM1. This STIM1–IP3R interaction, promoted by PC1, acts to suppress Ca^+2^ release; this regulation is mediated via the PI3K/Akt signaling pathway [49]. Moreover, the extracellular C-terminal domain of PC1 might act as a dominant-negative regulator, further enhancing Ca^+2^ release in *Pkd1*-mutant cells, underscoring the complex regulatory role of PC1 in intracellular Ca^+2^ homeostasis. Moreover, cells expressing the C-terminal fragment of PC1 exhibited elevated basal Ca^+2^ levels along with enhanced cell proliferation, suggesting a potential role of this domain in dysregulating Ca^+2^ signaling and promoting proliferative activity [51].

On the other hand, Ca^+2^ influx can also be mediated by other hetero-multimeric channels such as PC2/TRPC1 and PC2/TRPV4; these channels are similarly localized on the cell membrane and primary cilia, and they facilitate Ca^+2^ entry into cells in response to fluid flow. However, mutations in TRPC1 or TRPV4 alone do not lead to ADPKD, indicating that altered intracellular Ca^+2^ levels alone are not sufficient to initiate cyst formation; rather, the development of ADPKD appears to be specifically associated with functional impairments in either PC1 or PC2 [52]. Studies have identified calcium-activated chloride channels, such as Anoctamin 1 (TMEM16A), as key players in cystogenesis. Loss of functional PC1 has been shown to enhance TMEM16A activity, which disrupts normal calcium signaling and promotes increased cell proliferation and chloride secretion [53].

The Janus kinase and signal transducer and activator of transcription (JAK-STAT) pathway is another key signaling cascade involved in regulating cell proliferation, differentiation, and immune responses. JAK2 is highly expressed in renal cystic tubules during the early stages of ADPKD, where it contributes to the phosphorylation and activation of STAT3 [54]. PC1 can also activate STAT3 in a JAK2-dependent manner, promoting downstream transcriptional activity. However, defects in PC1, as observed in ADPKD, disrupt this regulatory mechanism, contributing to disease pathogenesis [55]. Furthermore; a variety of growth factors, such as epidermal growth factor (EGF), contribute to cystogenesis in ADPKD through stimulation of the proliferation of collecting duct cells, which are involved in cyst formation in ADPKD [56]. Inhibiting the EGF receptor (EGFR) has even been shown to decrease cystic growth in in vitro and in vivo models, suggesting that EGF signaling plays a significant role in promoting cyst development [57]. In addition, transforming growth factor alpha (TGF-alpha) is often found to be overexpressed in human polycystic kidneys. Nemo R., et al. reported that TGF-α can promote the abnormal proliferation of renal tubular epithelial cells, which is a key process in cyst formation and expansion. Overexpression of TGF-α in animal models leads to the development of cystic kidneys and accelerates the progression of the disease [58]. In addition, TGF-β is a major growth factor in ADPKD, and its upregulation is related to cyst expansion during disease progression [59]. Some studies indicate that TGF-β, particularly TGF-β2, might even have an inhibitory effect on cyst formation at earlier stages [60]. However, as the disease progresses, its role in promoting the deposition of extracellular matrix and fibrosis around the cysts becomes more prominent, contributing to cyst expansion and kidney tissue remodeling [60].

Both Hepatocyte Growth Factor (HGF) and its receptor, the tyrosine kinase receptor c-Met, are overexpressed in epithelial cells lining cysts in kidneys affected by ADPKD [61]. HGF is known to stimulate the formation of branched tubules in kidney development. In the context of ADPKD, this suggests that HGF signaling might contribute to the abnormal growth and branching of the kidney tubules, leading to cyst formation [62]. Research has shown that Insulin-like Growth Factor 1 (IGF-1) can increase the growth of cyst-lining epithelial cells by promoting cell proliferation [63]. Interestingly, this effect seems to be more pronounced in ADPKD cells compared to normal kidney cells since they express higher levels of IGF-1 receptors, making them more sensitive to IGF-1 stimulation [64]. This suggests that IGF-1 signaling could promote epithelial cell proliferation, leading to the expansion of cysts [63].

### 2.3. Fluid Secretion

**Figure 3:**
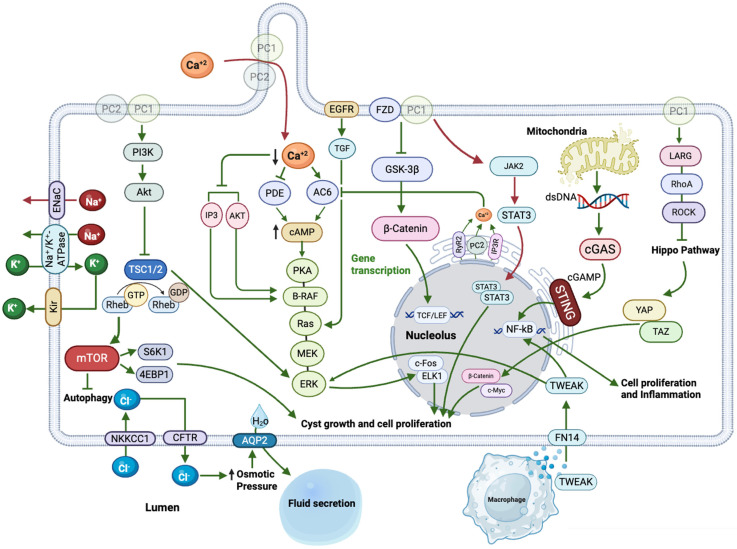
Intracellular signaling pathways driving renal cyst formation in Polycystic Kidney Disease (PKD). This schematic illustrates the disrupted signaling cascades resulting from mutations in Pkd1 and Pkd2 in PKD. Loss of functional PC1/PC2 reduces intracellular Ca^+2^ levels, leading to activation of AC6 and inhibition of PDE, which together elevate cAMP levels. Elevated cAMP activates PKA, which subsequently stimulates B-RAF, Ras, MEK, and ERK signaling cascades, promoting gene transcription, cell proliferation, and cyst growth. The Wnt/β–catenin pathway is activated through binding of Wnt ligands to the FZD receptor, resulting in inhibition of GSK-3β and stabilization of β-catenin, which translocate to the nucleus to induce transcription of proliferation-related genes. Dysregulation of this pathway contributes to epithelial cell proliferation in cyst-lining cells. The mTOR pathway is activated downstream of PI3K/Akt signaling. Akt inhibits the TSC1/2 complex, leading to activation of Rheb-GTP and mTOR. Active mTOR promotes protein synthesis via downstream targets such as S6K1 and 4EBP1, while also inhibiting autophagy. Concurrently elevated cAMP levels activate PKA, which stimulates the apical CFTR chloride channel, promoting Cl^−^ secretion into the cyst lumen. This chloride originates from basolateral uptake via NKCC1 and is supported by the Na^+^/K^+^–ATPase pump, which maintains ionic gradients. The efflux of Cl^−^ is followed by passive Na^+^ movement, regulated in part by ENaC, and water influx through AQP2, driven by the resulting osmotic gradient. Kir channels help maintain membrane potential to support continued ion transport. Loss of functional PC1 leads to aberrant activation of the JAK/STAT3 signaling pathway in cyst-lining epithelial cells. This activation promotes transcription of genes involved in cell proliferation, survival, and inflammation, contributing to cyst growth. Mitochondrial dysfunction in PKD leads to the release of mitochondrial dsDNA into the cytosol, where it is sensed by the cyclic GMP–AMP synthase (cGAS). Activation of cGAS triggers the STING pathway, which in turn activates NF-κB signaling. This cascade promotes the expression of pro-inflammatory cytokines, contributing to chronic inflammation and fibrotic remodeling in cystic kidneys. Loss of PC1 disrupts the activation of LARG, a Rho guanine nucleotide exchange factor, leading to hyperactivation of RhoA and its downstream effector ROCK. This dysregulation suppresses the Hippo pathway, resulting in nuclear accumulation of YAP/TAZ. Activated YAP/TAZ cooperates with β-catenin to upregulate c-Myc and other proliferative genes, driving abnormal epithelial cell proliferation and cyst growth in ADPKD. In the cystic microenvironment, infiltrating macrophages secrete the cytokine TWEAK, which binds to its receptor Fn14 on tubular epithelial cells. This interaction activates downstream NF-κB and ERK signaling pathways, promoting pro-inflammatory responses, epithelial cell proliferation, and cyst expansion. Red arrows indicate dysregulated pathways, and green arrows indicate activated pathways. Ca^+2^: calcium ion; PI3K: phosphoinositide 3-kinase; Akt/PKB: protein kinase B; TSC1/2: tuberous sclerosis complex 1/2; Rheb: Ras homolog enriched in brain; mTOR: mechanistic target of rapamycin; S6K1: ribosomal protein S6 kinase beta-1; 4EBP1: eukaryotic translation initiation factor 4E-binding protein 1; Cl^−^: chloride ion; NKCC1: Na^+^-K^+^-2Cl^−^ cotransporter 1; CFTR: cystic fibrosis transmembrane conductance regulator; Na^+^: sodium ion; K^+^: Potassium ion; ENaC: epithelial sodium channel; Kir: inward-rectifier potassium channel; Na^+^/K^+^-ATPase: sodium/potassium-transporting ATPase; AC6: adenylyl cyclase 6; PDE: phosphodiesterase; B-RAF: v-Raf murine sarcoma viral oncogene homolog B1; Ras: rat sarcoma viral oncogene homolog; MEK: mitogen-activated protein kinase; ERK: extracellular signal-regulated kinase; FZD: Frizzled receptor; GSK-3β: glycogen synthase kinase 3 beta; β-Catenin: beta-catenin; IP3: inositol 1,4,5-trisphosphate; IP3R: IP3 receptor; c-Fos: cellular proto-oncogene; ELK-1: ETS-like-1 transcription factor; TCF/LEF: T-cell factor/lymphoid enhancer factor; AQP2: aquaporin-2; STING: stimulator of interferon genes; JAK: Janus kinase; STAT3: signal transducer and activator of transcription 3; LARG: leukemia-associated Rho guanine nucleotide exchange factor; ROCK: Rho-associated protein kinase; YAP/TAZ: Yes-associated protein/transcriptional coactivator; TWEAK: TNF-like weak inducer of apoptosis; Fn14: fibroblast growth factor-inducible 14 receptor; NF-κB: Nuclear Factor kappa-light-chain-enhancer of activated B-cells; c-Myc: cellular Myelocytomatosis oncogene. Created with Biorender.com (accessed on 12 June 2025).

Although abnormal proliferation of epithelial cells is a major factor in cyst development, the active fluid secretion within the cysts plays a critical role in promoting cyst growth and is equally important in determining their size and impact on kidney function. A study focused on developing high-efficiency organoid models for ADPKD drug discovery highlights that the progressive formation of fluid-filled cysts is a hallmark of the disease, driven by abnormal signaling, increased cellular proliferation, and enhanced fluid secretion. This organoid model enables the in vitro investigation of these pathological processes [65]. The primary mechanism of fluid secretion and accumulation in renal cysts involves a cascade initiated by elevated cAMP levels in cyst-lining cells. This elevation results from dysregulated adenylyl cyclase (ACs) activity and inhibition of phosphodiesterases (PDEs), particularly in the absence of proper polycystin function. The increased cAMP activates PKA, which subsequently phosphorylates and stimulates the cystic fibrosis transmembrane conductance regulator (CFTR) chloride (Cl^−^) channels located on the apical membrane. As shown in Figure 3, chloride ions (Cl^−^) are secreted into the cyst lumen, initiating osmotic water influx that contributes to cyst growth. This process is driven by cAMP-mediated activation of basolateral potassium channels, which maintain the membrane potential necessary for sustained Cl^−^ secretion. Sodium ions (Na^+^) move passively through paracellular pathways or are absorbed via epithelial sodium channels (ENaC), balancing the charge. Water follows via aquaporin-2 channels [51,66]. Intracellular Cl^−^ levels are replenished by basolateral transporters such as NKCC1 and the Na^+^/K^+^–ATPase pump, maintaining electrochemical gradients. Overactivation of this ion transport machinery promotes cyst expansion and kidney damage [50].

### 2.4. Fibrosis

While fluid secretion and cell proliferation play a central role in cyst enlargement, they are not the sole contributors to disease progression. As cysts grow due to fluid accumulation, parallel processes of extracellular matrix (ECM) remodeling are initiated, facilitating further cyst expansion and contributing to disease progression [67]. Tubulointerstitial fibrosis is a major factor correlating with the rate of progression to end-stage renal disease in ADPKD, which is similar to its role in other chronic kidney diseases [68]. Fibrosis is characterized by the excessive accumulation of ECM proteins in the kidney tissue. In ADPKD, this leads to the replacement of functional kidney tissue with scar tissue, impairing kidney function [69]. While the mechanisms are still being explored, studies have shown that fibrosis in ADPKD shares some features with fibrosis in other kidney diseases, such as increased interstitial collagens, changes in metalloproteinases, and overexpression of TGF-β [70]. However, it also highlights unique, stage-specific aspects of ADPKD-related fibrosis. The abnormal accumulation of ECM can alter the behavior of kidney cells, including cyst-lining epithelial cells, through changes in cell–matrix interactions [71]. The increased expression and altered composition of ECM-modifying enzymes and components contribute to the pathogenesis of ADPKD. This includes elevated levels of structural proteins such as collagens I and III, basement membrane components like laminins B1 and B2, matricellular proteins including periostin and lumican, and ECM-regulating factors such as matrix metalloproteinase-2 (MMP-2) and transforming growth factor-beta (TGF-β) [67]. Furthermore, the enhanced interactions between ECM proteins and integrin receptors, like laminin-332, lead to abnormal cell proliferation. The binding of periostin to integrins activates signaling pathways (including integrin-linked kinase) that promote not only further cyst growth, but also ECM synthesis and tissue fibrosis. The upregulation of MMP-2 and TGF-β suggests an imbalance in ECM remodeling, further contributing to the disease process [72].

Furthermore, myofibroblasts, which are specialized cells capable of generating strong contractile forces and secreting large quantities of ECM, play a pivotal role in both wound healing and the advancement of tubulointerstitial fibrosis [70]. In ADPKD, the presence of α-smooth muscle actin (αSMA) provides a hallmark of these activated myofibroblasts, in which αSMA integrates into stress fibers and enhances their contractile capability [73]. These cells are frequently observed adjacent to expanding cysts, where elevated αSMA expression is accompanied by a pronounced increase in ECM components, leading to progressive fibrosis, tubular and nephron loss, reduced glomerular filtration rate, and a trajectory toward end-stage renal disease [74,75,76]. Immuno-depletion studies have further advanced our understanding of myofibroblasts’ origin and function in PKD. These investigations reveal that myofibroblasts derive primarily from the proliferation of resident interstitial cells, such as fibroblasts and pericytes, although a minor fraction arises from circulating bone-marrow-derived precursors [77]. Depletion studies using αSMA-targeted approaches reduce both renal fibrosis and cyst expansion, underscoring the direct involvement of myofibroblasts in the disease process [71]. This dual origin, local and systemic, reinforces the complexity of fibrotic progression in PKD and suggests potential therapeutic avenues targeting myofibroblast activation or recruitment.

Macrophages are pivotal immune cells involved in kidney development, acute and chronic renal injury, transplant responses, and repair processes [78,79,80]. Their diverse roles stem from their high functional plasticity, enabling them to adopt various activation states in response to environmental cues [81]. In renal fibrosis, macrophages contribute through several interconnected mechanisms. They secrete pro-fibrotic mediators that drive myofibroblast activation and persistence [82]. For instance, galectin-3 promotes a fibrogenic macrophage phenotype and directly stimulates myofibroblast differentiation and collagen synthesis. Macrophage-derived TGF-β and its activator thrombospondin (TSP), as well as IGF-1 and PDGF, enhance myofibroblast survival and proliferation. PDGF also targets renal pericytes and mesangial cells, further advancing fibrosis. Macrophages influence ECM remodeling through the secretion of MMPs and their tissue inhibitors of metalloproteinases (TIMPs) [83,84]. These enzymes support macrophage migration through tissues and facilitate ECM degradation. MMP-9 and other MMPs can also induce epithelial-to-mesenchymal transition (EMT), disrupting the tubular basement membrane and promoting interstitial fibrosis. Importantly, macrophages may also possess antifibrotic properties [85]. Experimental macrophage depletion during the tissue repair phase can lead to persistent scarring and myofibroblast retention, suggesting a role in resolving fibrosis [86]. M1 macrophages can induce mesangial cell apoptosis via tumor necrosis factor (TNF-α) and nitric oxide, while their interaction with apoptotic cells inhibits further inflammatory signaling [87,88]. Additionally, macrophages may induce myofibroblast apoptosis and degrade ECM, potentially destabilizing fibrotic tissue [89]. Collectively, the evidence indicates that distinct macrophage subsets may differentially regulate fibrotic processes by producing either matrix-depositing or matrix-degrading factors; however, this paradigm remains insufficiently investigated in the context of PKD.

TGF-β is abundantly expressed in cyst-lining epithelial cells across human, rat, and mouse models of PKD [75]. Activin A, a member of the TGF-β superfamily, plays critical roles in kidney development and repair, including inhibition of ureteric bud branching and participation in regenerative responses following injury. Elevated activin A expression in PKD models [75], along with findings that blockade of activin signaling attenuates cyst formation, highlights its potential involvement in cyst progression and positions it as a promising therapeutic target in renal cystic disease [90]. Activin A expression is also linked to conditions characterized by accelerated cyst formation due to cilia or polycystin deficiency, such as following renal injury or during early developmental stages. These findings suggest that activin A plays a pivotal role in cyst expansion and the progression to ESRD, given its involvement in epithelial repair and its classification within the TGF-β superfamily, known for promoting ECM gene expression in kidney cells [91]. Overall, the upregulation of various pro-fibrotic growth factors in PKD implies a coordinated cytokine-driven response that promotes ECM accumulation and contributes to disease progression.

### 2.5. Apoptosis

**Figure 4:**
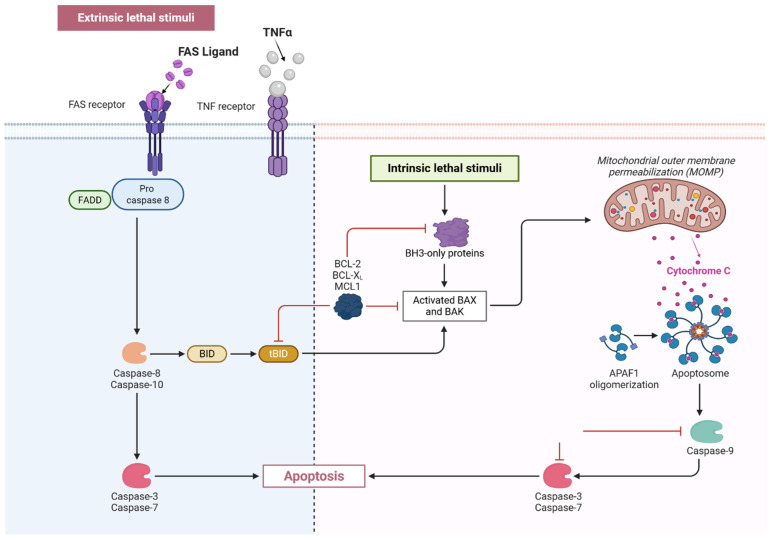
Schematic representation of intrinsic and extrinsic apoptotic pathways in Polycystic Kidney Disease (PKD). The extrinsic pathway is initiated by ligand binding to death receptors such as Fas or TNF receptor on the cell membrane, leading to caspase-8 activation. The intrinsic (mitochondrial) pathway is triggered by cellular stress, mitochondrial dysfunction, or DNA damage, resulting in BAX/BAK-mediated mitochondrial outer membrane permeabilization (MOMP), release of cytochrome c, and caspase-9 activation. Both pathways converge on caspase-3 activation, leading to apoptotic cell death. In PKD, dysregulated apoptosis contributes to cyst epithelial cell turnover and disease progression. BAX: Bcl-2-associated X protein; BAK: Bcl-2 antagonist/killer; BCL-2: B-cell lymphoma 2; BCL-XL: B-cell lymphoma-extra-large; MCL-1: myeloid cell leukemia-1; TNF-α:tumor necrosis factor-alpha; APAF-1: apoptotic protease-activating factor-1; BID: BH3-interacting domain death agonist; tBID: truncated BID; FADD: Fas-associated death domain protein. Created with Biorender.com (accessed on 12 June 2025).

Apoptosis, or programmed cell death, has been observed in the epithelial cells lining the cysts in both animal models and human ADPKD kidneys. Research has shown that epithelial cells lining renal cysts in ADPKD patients exhibit elevated rates of apoptosis compared to normal renal tissue [92]. Among the molecular mechanisms that regulate apoptosis, the caspase family of cysteine proteases plays essential roles in programmed cell death [93]. Both the intrinsic and extrinsic apoptosis pathways cooperate to stimulate the activity of the executioner caspases, which are then responsible for the events characteristic of apoptosis, including DNA fragmentation, protein degradation, and cell disintegration [94]. The intrinsic (mitochondrial) pathway is triggered by cellular stressors such as DNA damage, oxidative stress, or mitochondrial dysfunction [95,96]. These signals cause mitochondrial outer membrane permeabilization via BAX and BAK, leading to the release of cytochrome c, which activates adaptor protein apoptotic protease activating factor 1 (APAF1) and procaspase-9 to form the apoptosome [97]. This complex then activates caspase-9, which subsequently triggers executioner caspases, particularly caspase-3 and -7, leading to cell death via chromatin condensation, membrane blebbing, and apoptotic body formation [98,99] (Figure 4). In PKD, mitochondrial dysfunction is exacerbated by ROS overproduction and altered metabolism, sensitizing cyst-lining epithelial cells to apoptosis [100]. This is further supported by upregulation of pro-death mediators, including Bcl-2-associated X protein and Bcl-2 antagonist/killer, alongside downregulation of anti-apoptotic factors such as B-cell lymphoma 2 (BCL-2), B-cell lymphoma-extra-large, and myeloid cell leukemia-1 [92].

The extrinsic pathway is activated by ligand binding to death receptors such as Fas and tumor necrosis factor receptor (TNFR), initiating caspase-8 activation via adaptor proteins like Fas-associated death domain (FADD) [47,96]. Caspase-8 can directly activate executioner caspases or cleave BH3-interacting domain (BID) into truncated form (tBid), which amplifies the intrinsic pathway by promoting mitochondrial disruption [101] (Figure 4). In *Pkd2^+/−^* mice, components of the extrinsic pathway are upregulated, possibly due to chronic inflammation and increased levels of pro-inflammatory cytokines like TNF-α [102]. Elevated expression of Fas and Fas ligand has been reported in cystic kidneys, suggesting enhanced sensitivity to death-receptor-mediated apoptosis. This crosstalk between extrinsic and intrinsic pathways may lead to excessive apoptosis in renal tubular epithelial cells, contributing to tissue remodeling and cyst expansion [103].

### 2.6. Oxidative Stress and Mitochondrial Dysfunction

Oxidative stress and mitochondrial dysfunction are key pathophysiological drivers in the progression of ADPKD. Oxidative stress arises from an imbalance between the production of ROS and the antioxidant defense mechanisms [104]. ROS, including superoxide (O_2_^−^), hydrogen peroxide (H_2_O_2_), and hydroxyl radicals, are primarily generated in the mitochondria during aerobic respiration [105]. While low levels of ROS play physiological roles in cellular signaling, excessive accumulation causes damage to lipids, proteins, DNA, and cellular organelles, exacerbating cyst growth and renal fibrosis [106]. 

In PKD, both tubular epithelial and vascular endothelial cells in cystic kidneys exhibit elevated ROS levels [107]. A major source of this ROS is mitochondrial respiratory dysfunction, coupled with increased activity of NADPH oxidase enzymes, particularly NOX4. NOX4 is markedly upregulated in cyst-lining epithelial cells and is responsible for a significant portion of ROS generation [108]. This ROS overload not only damages the surrounding tissue but also activates further ROS production in a feedforward mechanism termed “ROS-induced ROS release”, which amplifies oxidative injury and promotes cystogenesis [104,109]. In PCK rats (a model for Polycystic Kidney Disease), elevated markers of oxidative DNA damage were detected early in both cyst-lining tubular epithelial cells (TECs) and non-cystic tubules. There was a marked increase in NOX4 expression and mitochondrial structural damage manifested as fragmented cristae, reduced mitochondrial content and respiration, and ROS overproduction. In endothelial cells, this led to impaired eNOS expression, capillary rarefaction, and worsening renal pathology [107]. Mitochondrial dysfunction in PKD extends beyond overproduction of ROS. Structural abnormalities such as fragmented mitochondria, swollen cristae, and reduced oxidative phosphorylation are common in cystic epithelial cells [110]. These cells undergo a shift from oxidative metabolism to glycolysis, which is a phenomenon similar to the Warburg effect observed in cancer cells [111]. This metabolic reprogramming provides rapid energy and biosynthetic intermediates that sustain the high proliferative state of cyst-lining cells. Moreover, impaired mitophagy, the selective degradation of damaged mitochondria, leads to the accumulation of dysfunctional mitochondria, further perpetuating oxidative stress and metabolic dysfunction [112].

The downstream consequences of oxidative stress are evident in multiple nephron segments, where ROS disrupt both transcellular and paracellular transport pathways [105]. In the thick ascending limb (TAL), ROS significantly enhances NaCl reabsorption by upregulating NKCC2 [113]. Sodium is then transported out of the cell by basolateral Na^+^/K^+^-ATPase, while chloride exits through Cl^−^ channels or KCl cotransport. Elevated luminal flow and stimulation by angiotensin II (ANG II), both of which are heightened in PKD, activate NOX4 and stimulate NKCC2 activity, amplifying sodium retention [114,115]. While the role of ROS in transcellular NaCl handling is well-documented, their influence on the paracellular movement of divalent cations such as magnesium (Mg^+2^) and Ca^+2^ remains poorly understood [105].

In the collecting duct, ROS stimulates ENaC, promoting Na^+^ reabsorption and K^+^ secretion in principal cells [116,117,118]. This process is augmented by aldosterone and the electrochemical gradient generated by Na^+^/K^+^-ATPase [119]. Vasopressin, another critical hormone in water homeostasis, further promotes water reabsorption by enhancing aquaporin-2 insertion into the luminal membrane. Elevated ROS levels, particularly under the influence of the renin–angiotensin–aldosterone system (RAAS), contribute to abnormal ENaC activation, leading to fluid retention and hypertension, which are hallmark features of progressive PKD [116]. The macula densa, a specialized cluster of cells at the junction of the TAL and distal convoluted tubule, also plays a critical role in ROS-mediated regulation of renal hemodynamics. This structure senses luminal NaCl and adjusts glomerular filtration rate (GFR) via tubuloglomerular feedback (TGF) [120]. Both NOX2 and NOX4 are expressed in the macula densa and contribute to O_2_^−^ generation [121]. ROS constrict the afferent arteriole directly and indirectly by scavenging nitric oxide (NO), thereby enhancing vasoconstriction and modulating renal blood flow [122]. Although the contribution of ROS to TGF is established, the precise ionic transport mechanisms that initiate this response remain incompletely characterized. Additionally, oxidative stress promotes cellular senescence, a state of irreversible growth arrest characterized by mitochondrial swelling, chromatin fragmentation, and inflammatory cytokine release [123]. In PKD, senescent cells accumulate in the renal parenchyma and contribute to chronic inflammation, fibrosis, and tubular atrophy. Impaired mitophagy exacerbates this by allowing damaged mitochondria to persist, increasing intracellular ROS, and activating stress-induced signaling pathways [124]. ROS also disrupts ER function, leading to protein misfolding, ER stress, and further activation of apoptosis or senescence pathways [125,126]. In summary, oxidative stress and mitochondrial dysfunction are deeply intertwined with the progression of PKD. They drive cyst expansion, alter renal electrolyte handling, promote cellular senescence, and induce fibrosis. These mechanisms present potential therapeutic targets; antioxidant therapies, mitochondrial protectants, and modulators of cellular metabolism are currently under investigation to slow disease progression and preserve renal function in PKD patients. The key molecular pathways implicated in ADPKD pathogenesis across different disease stages are summarized in Table 1.

## 3. Molecular Mechanisms’ Contributions to PKD Pathogenesis

The pathogenesis of ADPKD is driven by a complex interplay of molecular signaling pathways that regulate cell proliferation, fluid secretion, metabolism, and tubular architecture. Among the most studied mechanisms are aberrations in cAMP signaling, hyperactivation of the mTOR pathway, and dysregulation of the Wnt signaling network. In addition, emerging evidence highlights the involvement of novel signaling pathways in ADPKD. The STING pathway contributes to renal inflammation and fibrosis by activating innate immune responses. The TWEAK/Fn14 pathway promotes pro-inflammatory and pro-apoptotic effects in tubular epithelial cells. Meanwhile, the Hippo signaling pathway, through dysregulated Yes-associated protein (YAP) and transcriptional co-activator with PDZ-binding motif (TAZ) activity, drives abnormal cell proliferation and cyst expansion. Each of these pathways contributes uniquely to cyst initiation, expansion, and progression. The following sections explore these signaling mechanisms in detail, providing a comprehensive understanding of their roles in ADPKD pathophysiology.

### 3.1. cAMP-Driven Mechanisms in ADPKD Pathogenesis

cAMP plays a pivotal role in regulating key cellular functions, including cell proliferation and fluid balance. In healthy kidney epithelial cells, cAMP suppresses the B-rapidly accelerated fibrosarcoma (B-Raf), mitogen-activated protein kinase (MEK), and the extracellular signal-regulated kinase (ERK) signaling cascade. However, in epithelial cells affected by ADPKD, cAMP activates this same pathway, contributing to abnormal cellular behavior [127]. This dysregulation is intricately linked to impaired Ca^+2^ signaling caused by mutations in the *PKD1* or *PKD2* genes. A decrease in intracellular Ca^+2^ levels leads to enhanced activity of Ca^+2^-inhibitable AC6, while simultaneously suppressing the function of Ca^2+^/calmodulin-dependent PDEs, which are enzymes responsible for cAMP degradation [128]. As a result, the imbalance between increased synthesis and impaired breakdown of cAMP contributes to its pathological accumulation in cystic renal epithelial cells. An increase in intracellular cAMP levels initiates a cascade of signaling events through the activation of several downstream effector proteins. This second messenger molecule exerts its effects by directly binding to specific cAMP-responsive proteins that regulate a wide array of essential cellular functions, including transcriptional control, energy metabolism, cellular differentiation, and proliferation. The key effectors of cAMP signaling include exchange proteins directly activated by cAMP (Epac1 and Epac2), which are guanine nucleotide exchange factors that mediate cAMP-dependent but PKA-independent pathways [129,130]. The principal effector of cAMP signaling, however, is the cAMP-dependent protein kinase, commonly referred to PKA. Upon activation, PKA phosphorylates a wide range of substrates that modulate diverse physiological responses. In the context of ADPKD, aberrant cAMP signaling, particularly through PKA, has been shown to promote cyst growth by stimulating both epithelial cell proliferation and fluid secretion, thus contributing significantly to disease progression [131].

As shown in Figure 3, reduced intracellular Ca^2+^ in ADPKD disrupts normal suppression of the cAMP/PKA/B-Raf/MEK/ERK signaling cascade, leading to unchecked epithelial proliferation and cyst growth [45]. Under physiological conditions, Ca^2+^-activated AKT inhibits B-Raf, keeping MEK/ERK activity low. In ADPKD, this inhibition is lost, and elevated cAMP activates PKA, which directly stimulates B-Raf (but inhibits Raf-1), promoting ERK-mediated transcription of pro-proliferative genes [132,133]. Additionally, cAMP activates ion channels such as CFTR and potassium channels, driving electrolyte secretion and water influx into cysts, further contributing to cyst expansion [51]. The resulting fluid accumulation contributes to the progressive enlargement of the cyst (Figure 3). Recent research has further refined our knowledge of how this pathway contributes to cystogenesis and disease progression. Research involving transgenic mice expressing constitutively active B-Raf specifically in kidney collecting ducts revealed that such activation is sufficient to induce cyst formation in otherwise normal kidneys. These mice exhibited increased kidney weight, higher cyst number and size, elevated p-ERK levels, enhanced cell proliferation, immune cell infiltration, interstitial fibrosis, and a decline in kidney function. Furthermore, in mice with existing *Pkd1* mutations, active B-Raf expression accelerated disease progression [134]. A study demonstrated that Sorafenib, a Raf kinase inhibitor, effectively blocks cAMP-induced activation of the B-Raf/MEK/ERK pathway in human ADPKD cyst epithelial cells. At nanomolar concentrations, Sorafenib reduced ERK activity, inhibited cell proliferation stimulated by cAMP and EGF, and completely halted in vitro cyst growth in a three-dimensional collagen gel culture. These findings suggest that targeting B-Raf with small-molecule inhibitors like Sorafenib may be a viable therapeutic strategy to mitigate cyst expansion in ADPKD [135]. Wang et al. (2022) [136] identified the PKA-I regulatory subunit RIα as upregulated in ADPKD kidneys. Kidney-specific deletion of RIα increased PKA activity and worsened cystic disease, while genetic expression of a dominant-negative RIαB allele delayed cyst progression in *Pkd1^RC/RC^* mice. Additionally, the selective PKA catalytic inhibitor BLU2864 effectively suppressed cystogenesis both in vitro and in vivo without adverse effects, highlighting PKA-I inhibition as a promising therapeutic approach [136].

### 3.2. mTOR-Driven Mechanisms in ADPKD Pathogenesis

The mTOR pathway is critically involved in the pathogenesis of ADPKD. mTOR, a kinase that phosphorylates serine and threonine residues, assembles into two separate complexes: mTORC1 and mTORC2. mTORC1 regulates cell growth, proliferation, protein synthesis, and autophagy in response to nutrients, energy status, and growth factors [175]. In contrast, mTORC2 plays a pivotal role in regulating cytoskeletal dynamics, cell survival, and metabolism. It is activated by PI3K signaling or via inputs from the tuberous sclerosis complex (TSC1–TSC2). Once activated, mTORC2 phosphorylates and activates a subset of AGC family serine/threonine kinases, including Akt, serum- and glucocorticoid-regulated kinase 1 (SGK1), and protein kinase Cα (PKCα), thereby influencing multiple downstream pathways involved in cell survival and polarity [176]. Mutations in the PKD1 gene disrupt the interaction between PC1 and the tuberous sclerosis complex (TSC1/2), leading to aberrant activation of mTORC1 [137,177]. PC1 stabilizes the TSC1–TSC2 complex at the plasma membrane by protecting TSC2 from Akt-mediated phosphorylation and degradation, thereby maintaining TSC2’s inhibitory effect on mTORC1 [138,139]. Conversely, TSC2 is also necessary for proper localization of PC1 to the plasma membrane, indicating a bidirectional regulatory relationship. Loss of PC1 removes this inhibition, enabling Rheb-mediated activation of mTORC1. This results in phosphorylation of downstream targets such as ribosomal protein S6 kinase beta-1 (S6K1) and eukaryotic translation initiation factor 4E-binding protein 1 (4EBP1), promoting translation and cystic epithelial proliferation [140,141] (Figure 3). Additionally, mTORC1 inhibits autophagy via Unc-51-like kinase 1 (ULK1) suppression, a critical initiator of the autophagy cascade, further contributing to cyst growth and metabolic dysregulation. This cascade integrates with other pathways, such as PI3K/Akt and cAMP/PKA, to amplify proliferative and secretory signals in cyst-lining cells [142]. In the context of diseases like ADPKD, sustained mTORC1 activation leads to unchecked cell proliferation and impaired autophagy, both of which contribute to cyst expansion and kidney dysfunction.

Recent preclinical research comparing traditional and novel mTOR-targeting therapies has provided valuable insights into their effects on cystogenesis in ADPKD. In the Pkd1RC/RC mouse model, both sirolimus (mTORC1 inhibitor) and torin2 (dual mTORC1/2 inhibitor) effectively reduced cyst burden and improved renal function. In vitro, they suppressed phosphorylation of mTORC1/2 targets and decreased metabolic activity in Pkd1−/− cells. In vivo, both agents similarly inhibited S6, eukaryotic translation initiation factor 4E-binding protein 1 (4E-BP1), and Akt phosphorylation, and reduced proliferation in cystic kidneys [143]. Recent studies have further elucidated the role of mTOR signaling in ADPKD. For instance, research has demonstrated that mTORC1 activation can influence ciliary length and function, contributing to cyst formation. In a mouse model, the activation of mTORC1 led to elongation of primary cilia in renal epithelial cells, which is a change associated with early cystogenesis. Importantly, partial inhibition of mTORC1 in this model normalized ciliary length and reduced cyst development, highlighting the interplay between mTOR signaling and ciliary dynamics in ADPKD pathophysiology [144]. Therapeutically, mTOR inhibitors like sirolimus and everolimus reduce cyst growth in animal models but have shown limited success in clinical trials, likely due to subtherapeutic kidney levels or pathway compensation. To address these challenges, newer mTOR kinase inhibitors that target both mTORC1 and mTORC2 are under investigation. These inhibitors have demonstrated promising results in preclinical models by more effectively suppressing mTOR signaling and reducing cyst proliferation without the feedback activation commonly associated with rapamycin analogs [145].

### 3.3. Wnt-Driven Mechanisms in ADPKD Pathogenesis

The Wnt signaling pathway plays a pivotal role in the pathogenesis of ADPKD, with both its canonical and non-canonical branches contributing to cyst formation and progression. In the canonical Wnt/β-catenin pathway, Wnt ligands bind to Frizzled receptors and LRP5/6 co-receptors, enabling β-catenin to accumulate and relocate to the nucleus, where it regulates the expression of target genes promoting cell proliferation [146]. In ADPKD, loss of functional PC1 disrupts its inhibitory interaction with β-catenin, leading to unchecked Wnt/β-catenin signaling and increased proliferation in cyst-lining epithelial cells [147,148] (Figure 3). In *Pkd1*-deficient mice, aberrant expression of Wnt7a and Wnt7b was observed in cyst-lining cells, suggesting that upregulation of these ligands may initiate cystogenesis [61]. However, the role of canonical Wnt signaling in PKD is complex. Some studies have reported suppression of TCF/β-catenin activity in *Pkd1-* and *Pkd2*-mutant mice, suggesting that canonical Wnt signaling may not be universally upregulated in all PKD models. This discrepancy indicates that the involvement of canonical Wnt signaling in cystogenesis may be context-dependent, varying with disease stage or genetic background [149,150].

The non-canonical Wnt pathways, including the planar cell polarity (PCP) and Wnt/Ca^+2^ pathways, are also implicated in ADPKD. These pathways regulate cellular orientation and Ca^+2^ signaling, which are essential for maintaining tubular architecture and function. Disruption of PCP signaling impairs oriented cell division, leading to tubular dilation and cyst formation [20]. Moreover, Wnt ligands can associate with the external domain of PC1, influencing Ca^+2^ influx through PC2 channels. Mutations in *PKD1* or *PKD2* disrupt this Ca^+2^ signaling, further contributing to cystogenesis [151]. In *Pkd2*-deficient models, loss of Wnt-induced Ca^+2^ signaling and defects in cell migration were observed, underscoring the importance of non-canonical Wnt signaling in preventing cyst formation [152]. Studies have highlighted the complex interplay between Wnt signaling and other pathways in ADPKD. For instance, the loss of primary cilia not only activates canonical Wnt signaling but also disrupts PCP, underscoring the cilia’s role in modulating Wnt pathways [153]. Additionally, the interaction between Wnt signaling and the PI3K/AKT pathway has been observed, suggesting a broader network of signaling cascades contributing to cystogenesis [10]. Therapeutically, the Wnt signaling pathway represents a promising target for intervention in ADPKD. Inhibitors of the canonical Wnt/β-catenin pathway have shown encouraging results in preclinical studies, where they significantly mitigated cystogenesis in mouse models of the disease, pointing to their potential translational relevance [154]. Furthermore, pharmacological agents such as Endo-IWR1, which inhibit β-catenin activity by stabilizing the β-catenin destruction complex, have been demonstrated to reduce cyst growth and epithelial proliferation in ADPKD models [9]. However, given the pathway’s involvement in numerous physiological processes, careful modulation is necessary to avoid adverse effects.

### 3.4. STING-Driven Mechanisms in ADPKD Pathogenesis

The initial understanding of STING primarily revolved around its role in innate immunity, detecting cytosolic DNA (from viruses, bacteria, or damaged host cells) and initiating an interferon response [155]. However, its involvement in non-infectious chronic inflammatory diseases, including kidney diseases like ADPKD, has emerged as a major research focus. Recent studies have shed light on what activates STING in PKD. It is not typically viral or bacterial DNA. Instead, it is primarily the damaged DNA of host origin. Impairment of DNA repair processes has been linked to the development of ADPKD. Malfunctions in repair pathways like homologous recombination or non-homologous end joining can increase cellular vulnerability to genetic mutations and genomic instability, thereby accelerating disease progression [156,157]. Sustained activation of the DNA damage response may cause nuclear or mitochondrial DNA to escape into the cytoplasm. This aberrant presence of DNA is sensed by cyclic GMP–AMP synthase (cGAS), which subsequently activates STING, initiating downstream inflammatory signaling and contributing to disease pathology [158] (Figure 3). Recent findings have highlighted the role of the cGAS/STING pathway in promoting cystogenesis in ADPKD. Using immortalized murine collecting duct (IMCD) cells and conditional *Pkd1* knockout mouse models, researchers demonstrated that *Pkd1* deficiency induces elevated expression and nuclear translocation of cGAS, which activates pro-inflammatory signaling. This activation is associated with mitochondrial damage and genotoxic stress, which can be reversed by antioxidant treatment (MitoQ) or re-expression of PC1’s C-terminal tail. Inhibition or genetic deletion of cGAS significantly attenuates cyst development and preserves renal function in aggressive ADPKD models [159]. Moreover, the initial research identified STING’s influence on inflammation, fibrosis, and apoptosis, partly through Nuclear Factor kappa-enhancer of activated B-cells (NF-κB) activation. In *Pkd1*-mutant mouse models, STING expression is elevated in response to nuclear and mitochondrial DNA accumulation. Its activation drives disease progression by enhancing NF-κB signaling in mutant cells and promoting macrophage infiltration around cysts through increased TNF-α and monocyte chemoattractant protein-1 (MCP-1) expression. Notably, STING inhibition with C-176 delays cyst progression in both fast- and slow-progressing PKD models. It restores mitochondrial function, reduces micronuclei, promotes p53-mediated apoptosis, and reduces fibrosis, highlighting STING as a promising therapeutic target in ADPKD [160].

### 3.5. TWEAK/Fn14-Driven Mechanisms in ADPKD Pathogenesis

A significant recent advancement in understanding ADPKD progression has been the identification of the TNF superfamily cytokine TWEAK and its receptor Fn14 as key mediators of disease. They are potent mediators of inflammation, cell proliferation, tissue remodeling, fibrosis, and apoptosis, making them highly relevant to complex diseases like PKD [161]. A recent study solidified the critical role of this pathway, demonstrating its upregulation in polycystic kidneys and its direct contribution to cyst expansion, functional decline, and inflammation, thereby proposing it as a promising therapeutic target. It has been evident that the TWEAK/Fn14 signaling axis is aberrantly activated in ADPKD [162]. The analysis, encompassing both human ADPKD patient samples and an orthologous mouse model, revealed a consistent overexpression of both TWEAK and its receptor Fn14 within affected kidney tissue. Notably, elevated TWEAK levels were also detected in the urine and cystic fluid of ADPKD patients, suggesting its potential as a disease biomarker. Crucially, the study elucidated the functional consequences of TWEAK/Fn14 pathway activation in ADPKD. Through in vivo experiments, the researchers demonstrated that exogenous administration of TWEAK significantly exacerbated the cystic phenotype in murine models. Conversely, and perhaps most importantly for therapeutic development, targeted inhibition of the TWEAK pathway using anti-TWEAK neutralizing antibodies yielded substantial benefits. Treatment with these antibodies markedly slowed cyst progression, preserved crucial kidney function (as evidenced by improved renal parameters), and extended survival in the ADPKD mouse models. The mechanisms underpinning the observed therapeutic effects of TWEAK blockade were also investigated. The authors revealed that the beneficial impact of anti-TWEAK antibodies was primarily linked to a reduction in abnormal cell proliferation, which is a hallmark of cystogenesis. This outcome was partly attributed to reduced cellular proliferation-related MAPK signaling. Furthermore, TWEAK inhibition attenuated inflammatory responses by decreasing the activation of the NF-κB pathway and indirectly reducing the recruitment of macrophages to the kidney, which are known contributors to ADPKD inflammation and fibrosis. While the study noted a slight reduction in fibrosis and apoptosis, the major impact was attributed to controlling cell proliferation and inflammation [162]. The robust preclinical efficacy of anti-TWEAK antibodies positions them as highly promising candidates for clinical translation in ADPKD, representing a critical next step in therapeutic development.

### 3.6. Hippo Signaling-Driven Mechanisms in ADPKD Pathogenesis

The Hippo signaling pathway is an evolutionarily conserved mechanism that controls organ growth, maintains tissue balance, and regulates both cell proliferation and apoptosis. Central to this pathway are the transcriptional co-activators YAP and transcriptional co-activator with PDZ-binding motif TAZ, which are typically inhibited through phosphorylation by upstream Hippo kinases (MST1/2 and LATS1/2). Inactivation of Hippo signaling leads to dephosphorylation and nuclear accumulation of YAP/TAZ, where they interact with TEA domain (TEAD) transcription factors to drive gene expression programs related to cell growth and survival [9,163]. In the context of ADPKD, aberrant activation of YAP/TAZ has emerged as a key contributor to cyst formation and expansion [9,164,165].

Mechanistically, loss of PC1 activates the RhoGEF–RhoA–ROCK pathway, disrupting cytoskeletal dynamics and inhibiting MST1/2–LATS1/2. This inactivation releases YAP/TAZ, allowing their nuclear translocation and upregulation of pro-proliferative genes like c-Myc, thereby promoting cyst growth (Figure 3). Importantly, pharmacological inhibition of RhoA/ROCK signaling using the ROCK inhibitor Y-27632 significantly reduced YAP nuclear translocation, lowered c-Myc expression, and attenuated cystogenesis both in vitro and in vivo [166]. Lee et al. uncovered a cooperative role of TAZ and β-catenin in promoting cystogenesis in *Pkd1*-deficient kidneys. Loss of PC1 disrupts its inhibitory interaction with TAZ, enabling TAZ to bind AXIN1, stabilize nuclear β-catenin, and co-activate c-Myc expression. Conditional Taz deletion in *Pkd1*-null mice reduced cyst growth and improved renal function, highlighting the PKD1–TAZ–β-catenin–c-Myc axis as a key therapeutic target in ADPKD [9]. The Hippo pathway represents a crucial signaling axis in PKD pathogenesis, and understanding its complex interplay with polycystins offers exciting opportunities for developing novel therapeutic strategies. 

A comparative analysis of signaling pathways and their therapeutic implications is presented in Table 2, while recent experimental studies investigating molecular mechanisms and therapeutic targets in ADPKD are summarized in Table 3.

## 4. Epigenetic Mechanisms in ADPKD

Epigenetics refers to alterations in gene expression that occur without changes to the underlying DNA sequence. These epigenetic modifications control gene activity during cytodifferentiation by managing when and where specific genes are expressed, and they help maintain these expression patterns during and after cell division [167,168]. Recent studies in molecular biology have shown that epigenetic modifications play a crucial role in the onset and progression of ADPKD [169]. These heritable, reversible changes in gene expression do not alter the DNA sequence but have a significant impact on disease characteristics such as cell proliferation, fluid secretion, and inflammation [170]. PKD epigenetic changes are primarily mediated through four key mechanisms: DNA methylation, histone modifications, regulation by non-coding RNAs (ncRNAs), and chromatin remodeling [171]. DNA methylation is known to silence gene expression and has been implicated in the downregulation of key cyst-suppressing genes, such as *PKD1* and *PKD2*, which are essential for maintaining renal tubular cell integrity. These abnormal methylation patterns in renal epithelial cells disrupt normal gene expression, leading to enhanced cell proliferation, impaired differentiation, and increased fluid secretion, all of which contribute to cystogenesis and disease progression [172]. Recent studies have identified specific methylation changes in ADPKD, including hypermethylation at the 3′ end of the *PKD1* gene, which creates an environment conducive to cyst formation [173]. Additionally, altered methylation patterns influence signaling pathways, such as the Wnt/β-catenin pathway, which further drives cystic cell proliferation [45,174]. Emerging research also suggests that environmental factors, such as diet or inflammation, may exacerbate these epigenetic changes, highlighting the interplay between genetics, epigenetics, and external influences in ADPKD progression [178]. Therapeutic strategies targeting DNA methylation are under investigation. For example, demethylating agents like 5-azacytidine have shown promise in preclinical models by reactivating silenced cyst-suppressing genes, potentially slowing cyst development. However, challenges remain in ensuring the specificity of these interventions to avoid off-target effects in non-diseased tissues [179]. Histone modifications, such as acetylation and methylation, regulate chromatin structure and gene expression in ADPKD, influencing inflammation, fibrosis, fluid secretion, and cell proliferation [180]. Histone deacetylase inhibitors (HDACi), like trichostatin A, show promise in preclinical models by reducing cyst growth and preserving renal function by enhancing acetylation and suppressing CFTR-mediated fluid secretion [181]. BET inhibitors targeting acetylated histones also hold potential for mitigating inflammation and fibrosis [182]. Ongoing research explores combining histone-targeted therapies with epigenome-editing tools, like CRISPR, for precise ADPKD treatments [183]. Additionally, chromatin remodeling further modulates gene expression in ADPKD by altering the three-dimensional structure of the genome. Dysregulation in chromatin dynamics may influence the transcriptional activity of genes like PKD1 and Mucin-like protocadherin (MUPCDH), which is critical for epithelial integrity and cell polarity [184]. Recent studies suggest that dysregulated chromatin remodeling enhances the activity of pro-cystogenic pathways, such as mTOR and STAT3, by increasing the accessibility of their promoter regions, thus promoting cell proliferation and cyst expansion. For instance, altered SWI/SNF complex activity has been associated with the upregulation of two transcription factors known to drive cilia formation, Foxj1 and Rfx2, contributing to tissue remodeling in cystic kidneys [185]. Additionally, environmental stressors, such as hypoxia in cystic microenvironments, may exacerbate chromatin dysregulation, further disrupting epithelial homeostasis. The study by Grampp, Steffen, et al. shows that continuous cyst growth in ADPKD leads to regional tissue hypoxia in the kidneys. While it focuses on the upregulation of HIF-1α, it supports the idea that the cystic microenvironment, including hypoxia, can impact the behavior and homeostasis of epithelial cells in ADPKD [186]. Non-coding RNAs, particularly long non-coding RNAs (lncRNAs) and microRNAs (miRNAs), also contribute to ADPKD pathogenesis [187]. For example, miR-17~92 cluster upregulation has been associated with enhanced cell proliferation and cyst growth, while certain lncRNAs have been implicated in fibrosis and inflammation [188]. These molecules act by targeting mRNAs involved in cellular pathways such as mTOR, Wnt, and JAK/STAT, which are dysregulated in ADPKD [189,190]. In addition to classical epigenetic mechanisms, recent studies have identified novel genetic modifiers that influence ADPKD severity. For example, BICC1, an RNA-binding protein, was shown to directly interact with PC1 and PC2, accelerating cyst formation in both in vitro and in vivo models. Patient-derived data further support BICC1’s role in modulating early-onset ADPKD, emphasizing the significance of post-transcriptional regulation in disease progression [191].

## 5. Role of Emerging Biomarkers in the Pathogenesis of ADPKD

In recent years, the search for reliable biomarkers has become a critical aspect of ADPKD research, aiming to enhance early diagnosis, predict disease progression, and uncover novel therapeutic targets [192,193]. Several molecular markers have emerged as important players in the pathogenesis of ADPKD, not only serving as indicators of disease activity but also actively participating in the mechanisms underlying cystogenesis, cellular proliferation, fibrosis, and inflammation [192]. Among these, GPRC5A, Notch2, TBK1, and EZH2 have shown significant promise. GPRC5A is a retinoic acid-inducible G protein-coupled receptor that is increasingly recognized for its role in epithelial cell regulation. In ADPKD, GPRC5A is overexpressed in cyst-lining epithelial cells, particularly during the early stages of cyst development. This suggests a role in initiating and maintaining cystic expansion by influencing epithelial polarity, proliferation, and differentiation [194]. Mechanistically, GPRC5A is thought to modulate intracellular signaling pathways such as cAMP, Wnt/β-catenin, and MAPK, which are known to be dysregulated in ADPKD [154,195]. Its enhanced expression has also been associated with increased responsiveness to fluid secretion stimuli, contributing to cyst growth. Given its epithelial specificity and accessibility, GPRC5A represents a promising biomarker for both diagnostic imaging and targeted therapy. The Notch signaling pathway is essential for cell fate determination, proliferation, and differentiation during development. Among the Notch family members, Notch2 has been found to be abnormally activated in the kidneys of ADPKD patients and animal models [196]. This aberrant activation is closely linked to the dedifferentiation of tubular epithelial cells, increased proliferative signaling, and suppression of normal nephron maturation. Notch2 signaling has also been shown to interact with the mTOR and JAK/STAT pathways, further amplifying cystic growth and fibrotic responses [20]. Pharmacologic or genetic inhibition of Notch2 in experimental models leads to a reduction in cyst number and size, highlighting its functional relevance in disease pathogenesis [197]. As such, Notch2 is a compelling candidate for theragnostic applications, combining diagnostic biomarker potential with therapeutic value.

TANK-Binding Kinase 1 (TBK1) is a serine/threonine kinase that plays a central role in innate immunity, inflammation, and autophagy regulation. In the context of ADPKD, TBK1 is upregulated in cystic epithelial cells and interstitial compartments, where it contributes to abnormal inflammatory responses and altered vesicle trafficking. By phosphorylating downstream targets such as IRF3 and modulating NF-κB signaling, TBK1 amplifies a pro-inflammatory environment within the kidney [20]. This dual function, inflammation and autophagy dysregulation, links TBK1 to both cyst expansion and interstitial fibrosis, making it a strong mechanistic and prognostic biomarker. Targeting TBK1 may represent a novel therapeutic approach that addresses both the immune and metabolic dimensions of ADPKD pathogenesis.

Enhancer of Zeste Homolog 2 (EZH2) is a histone methyltransferase and a core component of the polycomb repressive complex 2 (PRC2), which mediates transcriptional silencing through trimethylation of histone H3 at lysine 27 (H3K27me3) [198]. In ADPKD, EZH2 is overexpressed in cystic epithelial cells and contributes to disease progression by repressing genes that control cell cycle arrest, differentiation, and apoptosis [199]. Functionally, EZH2 promotes a pro-proliferative and anti-differentiation transcriptional profile, driving unchecked growth of cystic epithelia. It also collaborates with other epigenetic regulators to sustain a repressive chromatin landscape, thereby reinforcing pathogenic gene expression programs. Inhibitors of EZH2 have shown efficacy in reducing cyst burden and slowing disease progression in preclinical models, underscoring its value as both a biomarker and therapeutic target [199].

## 6. Animal Models of Polycystic Kidney Disease

The study of PKD pathogenesis and the development of targeted therapies relies heavily on experimental models that accurately recapitulate the human disease. Among these, animal models have emerged as essential tools, offering in vivo systems for dissecting disease mechanisms and evaluating pharmacological interventions. Animal models enable investigation of complex, multicellular processes that are not replicable in vitro. In the context of PKD, where cyst initiation and expansion involve interactions among renal epithelial cells, vasculature, and extracellular matrix components, in vivo models provide critical mechanistic insight. Moreover, they facilitate longitudinal assessment of disease progression and therapeutic efficacy, which are imperative for translational research [200].

Complementing animal studies, in vitro models have become increasingly sophisticated and indispensable for studying PKD at the cellular and molecular levels. Traditional two-dimensional (2D) cultures of renal epithelial cells have been widely used to investigate gene function and signaling pathways implicated in cystogenesis. However, 2D cultures lack the three-dimensional architecture and microenvironmental cues critical for modeling the disease more realistically.

Recent years have seen significant advances in three-dimensional (3D) culture systems, which better recapitulate kidney tissue architecture and cyst formation. Human pluripotent stem cell (hPSC)-derived kidney organoids, for instance, can generate nephron-like structures exhibiting tubules and glomeruli, providing a physiologically relevant platform to study PKD pathogenesis. Unlike traditional immortalized cell lines or animal models, iPSC-derived organoids offer a unique advantage by incorporating patient-specific genetic backgrounds and a more faithful representation of human renal tissue architecture. Organoids generated from CRISPR-edited iPSCs harboring mutations in PKD1 or PKD2, as well as those derived from ADPKD patient samples, can spontaneously form cyst-like structures without the need for exogenous manipulation. These cysts resemble those observed in vivo in both morphology and function. For instance, kidney organoids exposed to forskolin, an agent that increases intracellular cAMP levels, demonstrate rapid cyst expansion mediated by CFTR-dependent chloride secretion, mimicking one of the core pathophysiological mechanisms in ADPKD [201,202]. Moreover, organoids derived specifically from collecting duct (CD) progenitors exhibit even more consistent cyst formation, making them particularly suitable for studying CD-specific contributions to cystogenesis. These CD organoids have been used to dissect downstream effects of PKD1 loss on cAMP signaling, calcium dysregulation, and cellular proliferation [203]. Organoids also respond predictably to pharmacological interventions, making them ideal for drug screening platforms. Indeed, recent studies have leveraged iPSC-derived ADPKD organoids in high-throughput compound screens. Fernandes et al. used *PKD1*-mutant organoids to screen a kinase inhibitor library and identified multiple compounds that suppressed cyst growth without affecting organoid viability [204]. Another study by Shimizu and colleagues identified retinoic acid receptor agonists as promising modulators of cyst expansion in CD-derived organoids [201]. These findings were further validated in mouse models, demonstrating the translational potential of this organoid-based screening approach. Subsequent work by Czerniecki et al. improved the maturation and reproducibility of these organoids, enhancing their utility in disease modeling [205]. Human iPSC-derived kidney organoids represent a transformative advance in ADPKD research. They offer a scalable, genetically tractable, and disease-relevant platform for modeling cyst initiation and expansion, elucidating signaling pathway disruptions, and testing candidate therapies. Their ability to mimic the complexity of the human kidney in a controlled setting makes them an indispensable tool for bridging the gap between bench research and clinical translation.

MDCK cells remain a widely used model for cyst formation due to their robust cystogenesis in 3D collagen or Matrigel matrices [206]. MDCK cysts have been extensively utilized to investigate signaling pathways involved in cyst expansion, including cAMP-mediated fluid secretion and mTOR activation [207]. Recent studies continue to use 3D cyst models for high-throughput drug screening to identify compounds that inhibit cyst growth [208]. Microfluidic “kidney-on-a-chip” platforms represent an exciting new frontier in PKD modeling. These systems recapitulate renal tubular flow dynamics and mechanical forces, providing physiologically relevant microenvironments absent in static cultures. For example, a human kidney-on-a-chip system capable of long-term culture of podocytes and tubular cells under fluid shear stress has been developed to allow detailed analysis of cellular responses to mechanical stimuli relevant in PKD [209]. More recently, these platforms have been adapted to model cystic epithelia derived from patient cells, offering personalized disease modeling and drug testing opportunities [210]. Further refinement of in vitro PKD models has been enabled by integrating CRISPR/Cas9 genome editing to generate isogenic cell lines and organoids with specific PKD mutations. This approach allows the dissection of genotype–phenotype relationships and mechanistic studies of disease progression [211].

Rodents—particularly mice and rats—are the predominant species used to model human diseases due to their genetic tractability, short breeding cycles, and physiological parallels to humans. The availability of advanced genome editing technologies (e.g., Cre-loxP and CRISPR/Cas9) has enabled the generation of precise genetic models, including both constitutive and conditional knockouts [212]. Furthermore, their small size and cost-efficiency make them ideal for preclinical studies requiring large cohorts. These models harbor mutations in genes orthologous to those causatives of autosomal dominant or recessive PKD in humans, including *PKD1, PKD2*, and *PKHD1*. Orthologous mouse models with targeted deletions in *Pkd1* or *Pkd2* recapitulate ADPKD pathology, though germline knockouts result in embryonic lethality. Conditional alleles have therefore been developed to allow spatiotemporal control of gene inactivation. Deletion of *Pkd1* in renal epithelial cells using KSP-Cre drives progressive postnatal cystogenesis [213]. Inducible systems such as Pax8-rtTA; TetO-Cre permit precise interrogation of disease onset and progression [214]. Targeting nephron progenitors with Six2-Cre reveals roles for *Pkd1* in early nephrogenesis. These models have enabled investigation of key pathogenic pathways, including cAMP, mTOR, and calcium signaling, and support the preclinical evaluation of candidate therapeutics such as tolvaptan [215]. In contrast, ARPKD has been modeled using the PCK rat, which carries a spontaneous mutation in *Pkhd1*. These animals develop both renal and hepatic cysts alongside progressive fibrosis, making them particularly suitable for studying hepatorenal manifestations and interstitial remodeling [216]. Non-orthologous and spontaneous models further extend the experimental toolkit. The Han:SPRD-Cy rat, although lacking a defined mutation in *Pkd1* or *Pkd2*, exhibits slowly progressive cyst formation and has been used extensively in long-term pharmacological and dietary intervention studies [217]. Similarly, the Jck mouse, with a mutation in the ciliary kinase gene *Nek8*, serves as a model for nephronophthisis-like ciliopathies and has facilitated high-throughput drug screening [218]. Mice deficient in the RNA-binding protein Bicc1 offer insights into post-transcriptional regulation in cystic disease [219]. Beyond rodents, alternative species provide complementary advantages for modeling PKD. Zebrafish enable rapid genetic screening and real-time imaging of cyst formation [220], while porcine models, given their physiological similarity to humans, are increasingly used to study disease progression and assess surgical or interventional approaches [221]. Together, these diverse in vivo systems represent a critical foundation for mechanistic discovery and therapeutic development in PKD research. Despite their utility, existing animal models have limitations that constrain translational applicability. Many rodent models exhibit more rapid cyst progression and lack the full spectrum of human disease features, such as variability in onset, vascular complications, or chronic pain [222]. Genetic redundancy and differences in nephron structure or renal physiology between species may also obscure human-relevant pathways. Moreover, some pharmacological responses in animal models do not fully predict clinical efficacy or toxicity [223]. These constraints highlight the need for complementary systems, including human organoids and large-animal models, to bridge the gap between experimental findings and patient outcomes.

## 7. Conclusions

ADPKD pathogenesis is driven by complex molecular interactions resulting from mutations in the PKD1 and PKD2 genes. These mutations disrupt calcium homeostasis and initiate a cascade of signaling abnormalities, including elevated cyclic AMP (cAMP) levels and activation of proliferative and secretory pathways such as mTOR, MAPK/ERK, and Wnt/β-catenin. Together, these signaling alterations drive the hallmark features of the disease, cyst initiation, epithelial cell hyperproliferation, fluid accumulation, and interstitial fibrosis. Although considerable progress has been made in mapping these pathways, integrating them into a cohesive therapeutic framework remains a challenge. Among the most promising therapeutic targets are the mTOR complexes (mTORC1/2). While mTOR inhibitors like sirolimus and everolimus showed efficacy in preclinical models, clinical trials revealed only modest benefits and notable side effects, underlining the importance of improved drug delivery methods and patient selection. MAPK/ERK pathway components, including B-Raf and MEK, have emerged as additional therapeutic targets. Inhibition of these kinases has reduced cystic growth in vitro and in animal models, although clinical translation is still under investigation. Furthermore, novel findings on the role of apoptosis, mitochondrial dysfunction, and extracellular matrix remodeling refine our understanding of cyst expansion and tissue scarring. Translating these insights into effective clinical interventions requires continued investigation into the cellular microenvironment, signal integration, and pharmacologic modulation. To move beyond a one-size-fits-all model, patient stratification based on biomarkers is urgently needed. Biomarkers such as serum bicarbonate, copeptin, and secreted Frizzled-related protein 4 (sFRP4) are being explored for their potential to predict disease progression and guide personalized therapy [7]. Stratifying patients by disease stage and dominant molecular mechanisms could allow for more tailored interventions, improving both efficacy and safety. Additionally, emerging therapeutic strategies, including modulation of glycosphingolipid metabolism and dietary/metabolic interventions such as calorie restriction, are being explored as complementary approaches and warrant further investigation in future studies. In summary, while the molecular landscape of ADPKD is complex, significant strides have been made in identifying key signaling pathways and therapeutic targets. A multi-targeted approach, combining pharmacologic agents that act on different pathways and supported by emerging biomarkers and delivery technologies, offers the most promising path forward. However, the true effectiveness of these emerging therapies remains uncertain due to the current lack of comprehensive clinical trials, which represents a key limitation of this review. Future research should focus on validating these approaches in clinical trials, refining patient selection strategies, and translating molecular insights into durable, effective treatments that can meaningfully improve patient outcomes.

## Figures and Tables

**Table 1:** Key Pathways by ADPKD Disease Stage.

Pathway	Disease Stage Involved	Functional Role in ADPKD	Evidence Strength	Ref(s)
JAK/STAT3	Cyst Growth	Promotes proliferation, survival, inflammation	Moderate	[54,55]
ROS/NOX4	All Stages	Drives fibrosis, apoptosis, senescence via oxidative damage	Strong	[101,102,103,104,105,106,107,108,109,110,111,112,113,114,115,116,117,118,119,120,121,122,123,124,125,126,127,128,129,130,131]
cAMP/PKA/ERK	Initiation, Cyst Growth	Promotes epithelial proliferation and fluid secretion	Strong (preclinical and clinical)	[132,133,134,135,136,137,138,139]
mTOR	Cyst Growth	Drives cell proliferation, inhibits autophagy	Strong (preclinical and clinical trials)	[140,141,142,143,144,145,146,147,148,149,150]
Wnt/β-catenin	Initiation, Cyst Growth	Enhances proliferation, disrupts polarity	Moderate (animal and organoid models)	[151,152,153,154,155,156,157,158,159,160,161,162]
STING	Cyst Growth, Fibrosis	Promotes inflammation and fibrosis via NF-κB	Emerging (preclinical)	[163,164,165,166,167,168]
TWEAK/Fn14	Cyst Growth, Fibrosis	Activates NF-κB and MAPK, increases proliferation and inflammation	Strong (mouse and human data)	[169,170]
Hippo/YAP/TAZ	Cyst Growth	Enhances proliferation via c-Myc with β-catenin cooperation	Emerging (mechanistic models)	[162,170,171,172,173,174]

cAMP: cyclic adenosine monophosphate; PKA: protein kinase A; ERK: extracellular signal-regulated kinase; mTOR: mammalian target of rapamycin; Wnt/β-catenin: Wingless/Integrated signaling pathway/β-catenin-dependent transcription; STING: stimulator of interferon genes; Hippo: Hippo signaling pathway; YAP: Yes-associated protein; TAZ: transcriptional coactivator with PDZ-binding motif; TWEAK: tumor necrosis factor-like weak inducer of apoptosis; Fn14: fibroblast growth factor-inducible 14; JAK: Janus kinase; STAT: signal transducer and activator of transcription; ROS: reactive oxygen species; NOX4: NADPH oxidase 4; NF-κB: nuclear factor kappa-light-chain-enhancer of activated B-cells.

**Table 2:** Comparative Summary of Key Signaling Pathways in ADPKD with Therapeutic Potential.

Pathway	Activation in ADPKD	Main Downstream Effects	Therapeutic Potential and Modulators	Ref(s)
JAK/STAT	↑ Activated	Proliferation, inflammation	JAK2 inhibitors (Preclinical)	[54,55]
ROS/NOX4	↑ Elevated	Apoptosis, fibrosis, mitochondrial dysfunction	MitoQ, antioxidants (In trials)	[101,102,103,104,105,106,107,108,109,110,111,112,113,114,115,116,117,118,119,120,121,122,123,124,125,126,127,128,129,130,131]
cAMP/PKA/ERK	↑ Upregulated	Cell proliferation, CFTR-mediated fluid secretion	Tolvaptan (Approved)	[132,133,134,135,136,137,138,139,175,176,177]
mTORC1/2	↑ Activated	Protein synthesis, cell growth, suppressed autophagy	Sirolimus, Torin2 (Trials/Preclinical)	[140,141,142,143,144,145,146,147,148,149,150]
Wnt/β-catenin	↑ Context-dependent	Proliferation, polarity loss, EMT	Wnt/β-catenin inhibitors (Preclinical)	[151,152,153,154,155,156,157,158,159,160,161,162]
STING	↑ Upregulated	NF-κB–driven inflammation, fibrosis	C-176, cGAS inhibitors (Preclinical)	[163,164,165,166,167,168]
Hippo/YAP/TAZ	↑ Nuclear accumulation	Drives c-Myc expression, proliferation	ROCK inhibitors (Y-27632) (Preclinical)	[162,170,171,172,173,174]
TWEAK/Fn14	↑ Overexpressed	MAPK/NF-κB activation, inflammation	Anti-TWEAK antibody (Preclinical)	[169,170]

cAMP: cyclic adenosine monophosphate; PKA: protein kinase A; ERK: extracellular signal-regulated kinase; mTORC1/2: mammalian target of rapamycin complex 1 and 2; Wnt/β-catenin: Wingless/Integrated signaling pathway/β-catenin-dependent transcription; STING: stimulator of interferon genes; Hippo: Hippo signaling pathway; YAP: Yes-associated protein; TAZ: transcriptional coactivator with PDZ-binding motif; TWEAK: tumor necrosis factor-like weak inducer of apoptosis; Fn14: fibroblast growth factor-inducible 14; JAK: Janus kinase; STAT: signal transducer and activator of transcription; ROS: reactive oxygen species; NOX4: NADPH oxidase 4; NF-κB: Nuclear Factor kappa-light-chain-enhancer of activated B-cells. ↑ = activated/upregulated in ADPKD.

**Table 3:** Summary of Key Experimental Studies in Autosomal Dominant Polycystic Kidney Disease (ADPKD).

Author(s)	Year	Species/System	PKD Gene/Pathway Targeted	Model Type/Intervention	Study Type	Key Observations/Comments
*Lee et al. [9]*	2020	Mouse	Wnt-β-catenin/TAZ	Endo-IWR1	*in vivo*	β-catenin inhibition reduces cyst growth and proliferation
*Shao et al. [24]*	2020	Mouse	*Ift88 + Pkd1*	*Tg737^orpk/orpk^* model	*in vivo*	Cilia loss impairs PC1/PC2 trafficking; double KO attenuates cystogenesis
*Cabrita et al. [53]*	2020	M1 cells	TMEM16A	PC1 loss/TMEM16A activation	*in vitro*	TMEM16A overactivity (due to PC1 loss) increases Ca^2+^ signaling and Cl^−^ secretion, promoting cystogenesis
*Patera et al. [54]*	2019	Mouse/human renal tubules	JAK2/STAT3	JAK2 expression and inhibition	*in vivo/ex vivo*	JAK2 is highly expressed in cystic tubules early in ADPKD; JAK2 inhibition reduces STAT3 activation and cyst growth
*Zhang et al. [59]*	2020	Mouse	TGF-β	TGF-β1 overexpression	*in vivo*	TGF-β1 promotes fibrosis and accelerates functional kidney decline in ADPKD
*Qin et al. [61]*	2012	Mouse	*Pkd1*	*Pkd1* KO	*in vivo*	β-catenin activation induces cysts; supports Wnt signaling
*Liu et al. [63]*	2013	Mouse	HGF, IGF-1	Transgenic overexpression	*in vivo*	IGF-1/HGF promote cyst-lining epithelial proliferation
*Dwivedi et al. [71]*	2023	Mouse	ECM/myofibroblasts	Myofibroblast depletion	*in vivo*	Depleting myofibroblasts reduces ECM deposition and cyst expansion, alleviating fibrosis
*Kim et al. [74]*	2022	Mouse	ECM/αSMA+ cells	ECM accumulation/cell–matrix interaction	*in vivo*	αSMA+ myofibroblasts near cysts drive fibrosis and nephron loss, progressing ADPKD
*Lee et al. [97]*	2024	Mouse/renal epithelial cells	Cytochrome C/Apoptosome	Oxidative stress/antioxidant modulation	*in vivo/in vitro*	Oxidative stress promotes apoptosome formation via cytochrome C/APAF1/caspase-9 pathway; antioxidants show protective potential
*Gonzalez-Vicente et al. [105]*	2019	Rat nephron segments	ROS	ROS modulation	*in vitro*	ROS impairs transcellular and paracellular ion transport across nephron segments, contributing to dysfunction
*Fedeles et al. [108]*	2024	Mouse	NOX4/ROS	Synthetic agent/ROS modulation	*in vivo*	NOX4 overexpression in cyst-lining cells enhances ROS; pro-oxidant agent promotes apoptosis of cystic cells and reduces cyst burden
*Klemens et al. [111]*	2025	Rat model of ARPKD	Glycolysis/metabolism	Cystic fluid metabolomics	*in vivo*	PKD cystic cells shift to glycolysis (Warburg effect), altering electrolyte and metabolite profile
*Haque & Ortiz [113]*	2019	Rat thick ascending limb (TAL)	Superoxide/NKCC2	PKC-mediated NKCC2 trafficking	*in vitro*	Superoxide upregulates NKCC2 activity via PKC, promoting sodium retention in PKD
*Zhang et al. [132]*	2021	Mouse	ERK/Cyclin-CDK	CDK1 activity assay	*in vivo*	ERK activation leads to CDK1 upregulation, driving cyst epithelial cell proliferation
*Parnell et al. [134]*	2022	Mouse (*Pkd1*-mutant)	B-Raf	Active B-Raf overexpression	*in vivo*	Active B-Raf expression in collecting ducts accelerates cyst growth in Pkd1-mutant mice
*Wang et al. [136]*	2022	Mouse/*PKD1*-mutant cells	PKA-RIα	BLU2864 inhibition	*in vivo/in vitro*	PKA-RIα promotes cystogenesis; BLU2864 suppresses effects
*Shillingford et al. [139]*	2006	Mouse	*Pkd1*	*Pkd1* KO	*in vivo*	mTORC1 upregulation; rapamycin reduces cyst expansion
*Holditch et al. [143]*	2019	Mouse/Pkd1−/− cells	mTOR	Torin2 vs. sirolimus	*in vivo/in vitro*	Both drugs reduce mTOR targets, proliferation, and metabolic activity
*Kim et al. [152]*	2016	Mouse	*Pkd2*/Wnt-Ca^+2^	Pkd2 KO	*in vivo*	Pkd2 loss impairs Wnt-Ca^+2^ signaling and cell migration; non-canonical Wnt is protective
*Lin et al. [153]*	2003	Mouse	*Kif3a + Pkd1*	*Pax8-Cre* conditional KO	*in vivo*	Loss of cilia promotes cysts; double KO surprisingly reduced cyst severity
*Li et al. [154]*	2018	Mouse	Wnt/β-catenin	Canonical Wnt inhibition	*in vivo*	Wnt/β-catenin inhibitors reduce cystogenesis
*Yoo et al. [159]*	2024	IMCD cells/mouse	cGAS/Pkd1	*cGAS* KO/MitoQ/PC1 tail	*in vivo/in vitro*	cGAS upregulated with *Pkd1* loss; promotes inflammation via mitochondrial/genotoxic stress. MitoQ or PC1-CTT reverses effects. cGAS inhibition reduces cyst growth and preserves renal function
*Wu et al. [160]*	2024	Mouse/renal epithelial cells	STING	*Pkd1* KO + STING inhibition (C-176)	*in vivo/in vitro*	Pharmacologic STING inhibition delays cystogenesis in both early and advanced ADPKD models; restores mitochondrial structure, reduces micronuclei formation, promotes p53-mediated apoptosis, and attenuates renal fibrosis
*Cordido et al. [162]*	2021	Mouse/Human	TWEAK/Fn14	TWEAK stimulation/anti-TWEAK antibody	*in vivo*/Human	TWEAK/Fn14 upregulated in ADPKD; anti-TWEAK Ab reduces cysts, inflammation, and preserves kidney function
*Cai et al. [166]*	2018	Mouse/renal cells	Hippo/YAP	ROCK inhibitor (Y-27632)	*in vivo/in vitro*	ROCK inhibition reduces YAP/TAZ activity and cysts

PKA: protein kinase A; ERK: extracellular signal-regulated kinase; Wnt/β-catenin: Wingless/Integrated signaling pathway/β-catenin-dependent transcription; STING: stimulator of interferon genes; Hippo: Hippo signaling pathway; YAP: Yes-associated protein; TAZ: transcriptional coactivator with PDZ-binding motif; TWEAK: tumor necrosis factor-like weak inducer of apoptosis; Fn14: fibroblast growth factor-inducible 14; JAK: Janus kinase; STAT: signal transducer and activator of transcription; ROS: reactive oxygen species; NOX4: NADPH oxidase 4; PKA-RIα: protein kinase A regulatory subunit I alpha; cGAS: cyclic GMP–AMP synthase; APAF1: apoptotic protease-activating factor 1; CDK1: cyclin-dependent kinase 1; B-Raf: v-Raf murine sarcoma viral oncogene homolog B; TMEM16A: transmembrane protein 16A; NKCC2: sodium–potassium–chloride cotransporter 2; TGF-β: transforming growth factor-beta; ECM: extracellular matrix; KO: knockout.

## Data Availability

No new data were created or analyzed in this study.

## Abbreviations

The following abbreviations are used in this manuscript:

ACs	Adenylate Cyclases
ADPKD	Autosomal Dominant Polycystic Kidney Disease
ANG II	Angiotensin II
ARPKD	Autosomal Recessive Polycystic Kidney Disease
Bak	Bcl-2 Antagonist/killer
Bax	Bcl-2-associated X Protein
BCL-2	B-cell Lymphoma 2
BCL-XL	B-cell Lymphoma-extra-large
B-Raf	B-Rapidly Accelerated Fibrosarcoma
Ca^+2^	Calcium
cAMP	Cyclic Adenosine Monophosphate
CD	Collecting Duct
c-Myc	Cellular Myelocytomatosis Oncogene
cGAS	Cyclic GMP–AMP Synthase
CFTR	Cystic Fibrosis Transmembrane Conductance Regulator
ECM	Extracellular Matrix
EMT	Epithelial-to-Mesenchymal Transition
ENaC	Epithelial Sodium Channel
eNOS	endothelial nitric oxide synthase
ER	Endoplasmic Reticulum
ERK	Extracellular Signal-Regulated Kinase
ESRD	End Stage Renal Disease
FPC	Fibrocystin
HGF	Hepatocyte Growth Factor
IGF-1	Insulin-like Growth Factor 1
IP3R	Inositol 1,4,5-Trisphosphate Receptor
JAK-STAT	Janus Kinase and Signal Transducer and Activator of Transcription
MCL-1	Myeloid Cell Leukemia-1
MDCK	Madin-Darby Canine Kidney
MEK	Mitogen-Activated Protein Kinase
MMP	Matrix Metalloproteinase
mTOR	Mammalian Target of Rapamycin
Na^+^	Sodium Ion
NADPH	Nicotinamide Adenine Dinucleotide Phosphate
NHKS	Normal Human Kidneys
NCCE	Non-Capacitative Ca^+2^ Entry
NO	Nitric Oxide
NOX4	NADPH Oxidase 4
PC1	Polycystin-1
PC2	Polycystin-2
PDE	Phosphodiesterases
PDGF	Platelet-Derived Growth Factor
PKA	Protein Kinase A
PKD	Polycystic Kidney Disease
ROS	Reactive Oxygen Species
RyR	Ryanodine Receptor
STIM1	Stromal Interaction Molecule 1
TGF-α/β	Transforming Growth Factor Alpha/Beta
TIMPs	Tissue Inhibitors of Metalloproteinases
TMEM16A	Transmembrane Member 16A Anoctamin 1
TRP	Transient Receptor Potential
TRPP	Transient Receptor Potential Polycystin
TSP	Thrombospondin
WGS	Whole-Genome Sequencing
